# Entropy engineering for efficient ionic thermoelectric conversion

**DOI:** 10.1093/nsr/nwag031

**Published:** 2026-01-16

**Authors:** Boyang Yu, Zhong Lin Wang, Jiangjiang Duan

**Affiliations:** Beijing Institute of Nanoenergy and Nanosystems, Chinese Academy of Sciences, China; School of Nanoscience and Engineering, University of Chinese Academy of Sciences, China; Beijing Institute of Nanoenergy and Nanosystems, Chinese Academy of Sciences, China; School of Nanoscience and Engineering, University of Chinese Academy of Sciences, China; School of Materials Science and Engineering, Georgia Institute of Technology, USA; Wuhan National Laboratory for Optoelectronics, Huazhong University of Science and Technology, China

## Abstract

Thermogalvanic technologies, as a type of ionic thermoelectrics, hold promise for waste-heat harvesting and cooling, and the entropy engineering is key to achieving high energy conversion efficiency, bringing both challenges and opportunities.

Over 60% of global energy consumption is ultimately rejected as heat [1], a trend further intensified by the rapid growth of artificial intelligence and consumer electronics. Despite the magnitude of this loss and its associated safety and thermal-management concerns, most waste heat remains difficult to harvest using conventional heat engines due to its widespread and near-room-temperature features. Recently, thermogalvanic technologies, a class of ionic thermoelectrics, have provoked intensive interest for their ability to directly and continuously convert heat with small temperature differences into electricity via reversible redox reactions (Fig. 1a) [2−5]. These systems offer appealing advantages in thermopower, cost, flexibility, and scalability; however, their inferior efficiency remains a critical challenge to practical deployment [6]. Fundamentally, thermogalvanic conversion originates from the temperature dependence of electrochemical potential (*E*), also named thermogalvanic effect, which is governed by the reaction entropy change (∆*S*_rxn_) and quantified by the thermogalvanic thermopower as: *S*_tg_ = −∆*S*_rxn_/*n*F (where *n* is the number of transferred electrons and *F* is the Faraday constant) [1]. Therefore, entropy engineering, encompassing but not limited to the regulation of ∆*S*_rxn_ through redox-couple chemistry and electrolyte/electrode design, is key to enhancing thermogalvanic thermopower and consequently improving efficiency (η ∝ *S*_tg_^2^). In this perspective, we highlight the fundamentals and advances in entropy engineering for the thermogalvanic effect, also discussing outstanding challenges and future directions.

**Figure 1.**
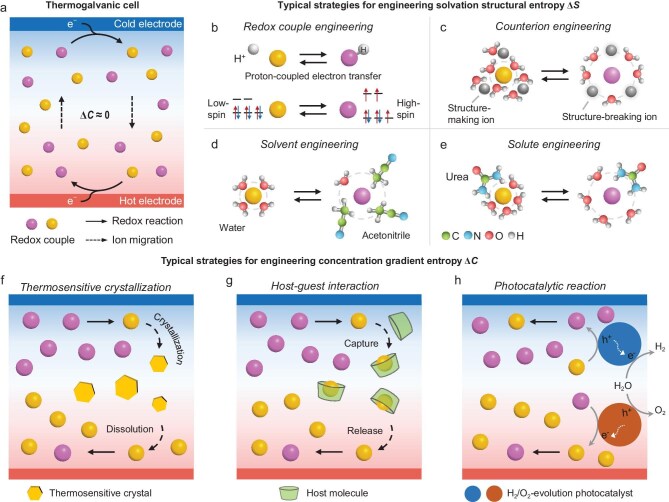
Schematic of entropy engineering for thermogalvanic conversion. (a) Schematic of a thermogalvanic cell. For a given redox couple, oxidation at the hot side results in a positive thermogalvanic thermopower (*S*_tg_), whereas oxidation at the cold side yields a negative *S*_tg_. (b–e) Schematic of four typical solvation structural entropy engineering strategies by (b) designing redox couples, and tuning their chemical surroundings: (c) counterions, (d) solvents, and (e) solute species. (f–h) Schematic of three typical concentration gradient entropy engineering strategies by (f) thermosensitive crystallization, (g) host–guest interaction, and (h) photocatalytic reaction.

According to thermodynamic theory, ∆*S*_rxn_ can be decomposed into multiple contributions, including solvation, concentration, configuration, phonon, and electron entropy terms. In most cases, the solvation structural entropy (∆*S*) is the dominant contributor, roughly exceeding the other terms by orders of magnitude. In detail, the solvation structure is governed not only by intrinsic properties of the redox couples (e.g. charge density, electron spin state, coordination environment, and proton-coupling), but also by their chemical surroundings, including counterions, solvents, and solute species. Accordingly, current representative strategies for engineering solvation structural entropy include: **(1) Designing new redox couples** (Fig. 1b). For instance, the proton-coupled electron transfer reaction between hydroquinone and benzoquinone delivers a *S*_tg_ of −4.29 mV K^−1^ [7]. For transition-metal complexes (e.g. Fe-, Ru-, and Co-based), a low-spin to high-spin state transition accompanied in the redox reaction can introduce an additional solvation-entropy contribution, providing a useful handle to design redox ions [8]. **(2) Engineering counterions** (Fig. 1c). A general trend observed in aqueous Fe(CN)_6_^3−/4−^ and Fe^3+/2+^ electrolytes is that structure-breaking counterions with weak ion-water interactions tend to promote a more disordered solvation shell and thus a slightly higher *S*_tg_, whereas structure-making counterions typically suppress *S*_tg_ [9]. **(3) Engineering solvents** (Fig. 1d). The *S*_tg_ of Fe^3+/2+^ depends strongly on solvent identity, reaching as high as −3.73 mV K^−1^ in acetonitrile and other nitrile solvents [10]. Likewise, the *S*_tg_ of I_3_^−^/I^−^ can vary by several-fold among water, organic solvents, and ionic liquids; notably, a comparatively large magnitude (ca. −0.9 mV K^−1^) has been reported at infinite dilution in aqueous solution [11]. By contrast, Fe(CN)_6_^3−/4−^ exhibits a much weaker solvent dependence, and solvent substitution alone fails to markedly enhance *S*_tg_ [12]. **(4) Introducing solute additives** (Fig. 1e). For instance, adding highly soluble amide derivatives (e.g. urea) and chaotropic species to aqueous Fe(CN)_6_^3−/4−^ electrolytes increases *S*_tg_ from 1.4 to 4.2 mV K^−1^ [4]. In parallel, molecular dynamics (MD) simulations provide a route to quantify solvation-entropy contributions; for selected systems, *S*_tg_ can be estimated by combining free-energy perturbation methods with thermodynamic analysis [13].

The concentration gradient entropy (∆*C*), arising from temperature-driven redistribution of redox species, has been derived and validated as another significant contributor to ∆*S*_rxn_ [2]. This term was long overlooked because it is negligible in traditional, compositionally uniform systems (Fig. 1a). According to the Nernst equation of *E*, ∆*C* depends alone on the differential concentration profiles of the oxidized (*O*) and reduced (*R*) species under a temperature gradient: ∆*C* ∝ (d*O*/d*T* − d*R*/d*T*). Notably, ∆*C* can exceed the intrinsic ∆*S*_rxn_ by several-fold and may carry an independent sign. Consequently, engineering ∆*C* can substantially enhance *S*_tg_ (up to 8.2 mV K^−1^) [3] and *η* (over 11% of the Carnot efficiency) [2], and even enables p–n type conversion in thermogalvanic systems [14]. There are three representative strategies to tune concentration gradient entropy: **(1) Thermosensitive crystallization** (Fig. 1f). Engineering of thermosensitive crystallization for redox ions is a paradigm-shift approach that leverages crystals with strong temperature-dependent solubility to establish an ionic concentration gradient between the cold and hot electrodes [2]. An expanding library of thermosensitive crystals has been developed for various redox couples (e.g. Fe(CN)_6_^3−/4−^, Fe^3+/2+^, I_3_^−^/I^−^, and Cu^2+/0^), and has been demonstrated to be effective not only in liquid electrolytes but also in quasi-solid state and even at subzero temperatures [5,15−17]. **(2) Host–guest interaction** (Fig. 1g). Given the pronounced hydrophilic-hydrophobic contrast of the I_3_^−^/I^−^couple, a host–guest molecular-recognition strategy has also been developed to selectively capture and release polyiodides upon temperature variation, thereby generating a sizable ∆*C* and *S*_tg_ (ca. −2 to −4 mV K^−1^) [18]. **(3) Photocatalytic reaction** (Fig. 1h). Coupling with photocatalysis is another route to amplify ∆*C* and *S*_tg_ in Fe(CN)_6_^3−/4−^ systems while enabling synergistic hydrogen production, where Fe(CN)_6_^3−^ is reduced by an O_2_-evolution photocatalyst at the hot electrode and Fe(CN)_6_^4−^ is oxidized by a H_2_-evolution photocatalyst at the cold electrode [3].

Although significant progress has been achieved in enhancing thermogalvanic thermopower through entropy engineering, several challenges remain in both theoretical prediction and comprehensive parameter optimization. On the one hand, current MD simulations are largely limited to selected redox species in dilute solutions, whereas high-concentration and quasi-solid systems electrolytes are being increasingly favored for practical thermogalvanic devices. It is therefore urgent to develop predictive frameworks that can reliably estimate *S*_tg_ and guide electrolyte/material design under realistic conditions. One promising direction is to integrate emerging computational approaches, such as AI and machine-learning-assisted modeling, with physics-based simulations to help bridge the gap between dilute-model systems and practical, highly concentrated electrolytes. In addition, it should be emphasized that the announcement of entropy engineering in thermogalvanic research must remain theoretically rigorous and quantitatively supported, rather than being invoked *post hoc* to rationalize anomalous voltage observations. Likewise, without identifying and substantiating the origins of the underlying entropy, the numeric ratio of an apparent device voltage to an imposed temperature difference (∆*V*/∆*T*) is thermodynamically meaningless and should not be equated with thermopower; such practice has often caused misleading reports on ionic thermoelectric conversion.

Before discussing comprehensive optimization of thermogalvanic parameters, it is necessary to clarify normalized evaluation standards and several common misconceptions:

**(i) Evaluating the energy conversion efficiency (*η*) of thermogalvanic systems.** It is widely accepted that *η* is co-determined by the *S*_tg_, electrical conductivity (*σ*), and thermal conductivity (*κ*), often described qualitatively as *η*∝(*S*_tg_^2^)*σ*/*κ*. While this classical relationship provides useful directional guidance for improving efficiency, it cannot quantify the actual energy conversion processes across diverse thermogalvanic devices. Specifically, the device electrical conductance (or effective *σ, σ*_eff_) reflects the combined effects of charge transfer, ion transport, and ohmic resistances [19]. Meanwhile, the device thermal conductance (or effective *κ, κ*_eff_) arises from thermal conduction and convection and even interfacial heat losses together. These complexities become even more pronounced for emerging architectures (e.g. flow cells and membrane-based cells) and working modes (e.g. battery-type and capacitor-type configurations), where the governing factors cannot be captured by bulk *σ* and *κ* alone. Therefore, directly calculating *η* or *ZT* (figure of merit) value based on the term (*S*_tg_^2^)*σ*/*κ* is inappropriate and generally incorrect for thermogalvanic systems. Instead, a universal efficiency definition based on energy balance is recommended: *η* = *Q*_electrical_/*Q*_heat_, where *Q*_electrical_ is the output electrical energy and *Q*_heat_ is the input heat energy. Experimentally, *η* can be directly obtained from steady-state discharge tests combined with heat-flux measurements. But for some atypical conditions, any additional energy inputs (e.g. solar, mechanical, electrical, or consumable chemical energy) must also be included in the energy accounting to analyze *η*.

**(ii) Evaluating the electrical power density (*P*) of thermogalvanic systems.** In addition to *η*, output power is critical for practical deployment and should be reported as a complementary performance metric. Although the output power can be directly measured, there remain several typical mistakes in reporting power density value in previous research. First, misemploying the smallest cross-sectional area among electrodes, cell chamber, and heat-transport pathways to calculate the *P* when these areas are not identical. Second, misemploying the area of a single cell to calculate the *P* of the whole integrated or stacked device. Third, confusing the contribution of Faradaic and non-Faradaic currents to output power.

**(iii) Comparison of *η* and *P* across different thermogalvanic systems.** In practice, operating conditions, particularly the hot temperature (*T*_hot_) and temperature difference (∆*T*), strongly affect both *η* and *P*. Accordingly, it is necessary to further analyze the Carnot-relative efficiency (*η*_r_ *=* *η*/*η*_Carnot_, where the *η*_Carnot_ is ∆*T*/*T*_hot_) and the temperature-normalized power density (*P*/∆*T*^2^). Notably, Carnot-relative efficiency is meaningful only for pure heat-to-electric conversion; if other energy sources contribute, they must be rigorously excluded to prevent unphysical or absurd results.

Ultimately, regardless of which metrics are adopted, the most important requirement is to thoroughly clarify the underlying energy conversion mechanism, the origin of performance improvements, as well as the complete energy flux in the system.

Simultaneous optimization of *S*_tg_, *σ*_eff_, and *κ*_eff_ is essential to achieve a substantial increase in *η* yet remains challenging because these parameters are strongly interdependent. For instance, regulating the solvation structure of redox ions using nonconductive solvents or solute additives will severely compromise *σ*_eff_, despite a possibly high *S*_tg_. Therefore, future efforts should focus on seeking effective entropy-engineering strategies that balance *S*_tg_, *σ*_eff_, and *κ*_eff_ rather than optimizing any single parameter in isolation. Concentration gradient entropy engineering offers promise in this regard; however, it is still constrained by saturation limits and restrictions in material choices. Moreover, pairing p-type and n-type thermogalvanic systems to construct integrated devices poses additional difficulties due to the Cannikin law governing overall performance, as well as practical issues of material compatibility (e.g. toxicity and stability). In particular, the relatively poor n-type thermogalvanic performance must be urgently improved, but progress is hindered by sluggish redox kinetics and the strong coupling between *S*_tg_ and redox-species concentration.

Overall, prior studies in entropy engineering have opened the door to efficient thermogalvanic conversion, and future breakthroughs are expected in the following directions:

**(i) Novel entropy-engineering mechanisms to achieve *S*_tg_ on the order of 10 mV K^−^^1^.** Current thermogalvanic systems deliver only several mV K^−1^, which remains insufficient for convenient device integration and small temperature difference harvesting (e.g. wearable electronics and environmental monitors). Although thermodiffusion and hybrid systems can exhibit seemingly high ionic thermopower, intrinsic limitations in efficiency and intermittent operating mode restrict their practical utility. Raising thermogalvanic thermopower toward tens of 10 mV K^−1^ would provide an attractive platform for higher efficiency and broader applications, and may be technically feasible through multiple entropy engineering. Achieving this goal requires revealing the underlying factors in each entropy terms of ∆*S*_rxn_, as well as leveraging AI-driven approaches to accelerate materials discovery and optimization.

**(ii) Synergistic entropy engineering across redox ions, electrode materials, and device architecture.** Previous strategies focused on singly tuning entropy of redox ions in electrolytes, but are challenged by strong coupling among *S*_tg_, *σ*_eff_, and *κ*_eff_. In contrast, rational electrode design may simultaneously enhance redox kinetics and increase ∆*S*_rxn_ by modulating configuration and phonon entropy, offering a potential route to improve both *S*_tg_ and *σ*_eff_ without compromising *κ*_eff_. In addition, device-architecture strategies that decouple and optimize electrical versus thermal transport can be pursued largely independently of electrolyte chemistry, and can be combined with entropy-engineered materials to enable synergistic optimization of *S*_tg_, *σ*_eff_, and *κ*_eff_.

**(iii) Scalable, replicable, and modular entropy-engineering strategies.** The economic viability of conventional technologies for harnessing low-temperature heat below 100°C remains debated [20], but underscores the primary principle of developing thermogalvanic technologies. First, it is unviable to pursue high thermogalvanic performance if sacrificing the inherent inexpensive and scalable merits. Second, future research needs to make efforts on scalable manufacturing of entropy-engineered materials and prototype devices, alongside improved operational reliability and durability. Third, integrating thermogalvanic conversion into existing commercial technologies may be an effective near-term route to real-world deployments with minimal cost.

**(iv) Expanding entropy engineering to applications beyond heat harvesting**, including thermogalvanic sensing, refrigeration, and energy storage. For example, quasi-solid thermogalvanic electrolytes show promise for constructing electronic skins for thermal perception, where high and stable *S*_tg_ with fast response and broad temperature span are required. For thermogalvanic refrigeration, large entropy change per unit mass of electrolyte can be more important than an apparently high *S*_tg_, requiring materials with low heat capacity and high solubility that enable large entropy modulation. Given peak–valley mismatches between electricity supply and demand, combining heat harvesting and energy storage within a single thermogalvanic platform is also compelling, which may be enabled by intercalation-type electrodes with configuration and phonon entropy engineering.

Furthermore, it is worth noting that heat is often parasitic to other energy processes, such as steam from power plants, warm cycling water from datacenters, energy dissipation from mechanical friction, and ambient energy from wind, solar, and ocean. Thermogalvanic technologies that enable hybrid energy conversion could therefore be particularly impactful in real-world deployments, which require a deeper understanding of coupled multiphysics effects on entropy engineering.
